# Using hydrodynamic focusing to predictably alter the diameter of synthetic silk fibers

**DOI:** 10.1371/journal.pone.0195522

**Published:** 2018-04-12

**Authors:** Bradley Hoffmann, Catherine Gruat-Henry, Pranothi Mulinti, Long Jiang, Benjamin D. Brooks, Amanda E. Brooks

**Affiliations:** 1 Department of Mechanical Engineering, North Dakota State University, Fargo, North Dakota, United States of America; 2 Department of Electrical Engineering, North Dakota State University, Fargo, North Dakota, United States of America; 3 Department of Pharmaceutical Sciences, North Dakota State University, Fargo, North Dakota, United States of America; University of Houston, UNITED STATES

## Abstract

Spiders and silkworms provide a model of superior processing for multifunctional and highly versatile high-performance fibers. Mimicking the spider’s complex control system for chemical and mechanical gradients has remained an ongoing obstacle for synthetic silk production. In this study, the use of hydrodynamic fluid focusing within a 3D printed biomimetic spinning system to recapitulate the biological spinneret is explored and shown to produce predictable, small diameter fibers. Mirroring in silico fluid flow simulations using a hydrodynamic microfluidic spinning technique, we have developed a model correlating spinning rates, solution viscosity and fiber diameter outputs that will significantly advance the field of synthetic silk fiber production. The use of hydrodynamic focusing to produce controlled output fiber diameter simulates the natural silk spinning process and continues to build upon a 3D printed biomimetic spinning platform.

## Introduction

Orb-weaving spiders, such as the golden orb-weaver, *Nephila clavipes*, produce up to 6 solid silk fibers, each with a specified ecological purpose. Major ampullate (MA) silk fibers, the most studied of the group, possess both high strength and elasticity [1,2]. Despite our ability to reproduce and manipulate the key genetic elements of major ampullate silk proteins, the unique composition and mechanical balance of the fiber remains enigmatic and unrivalled by man-made materials [3–5]. Limitations in the ability to capture both the genetic elements of the silk proteins and the complexity of the spider’s spinning system have made capitalizing on silk’s properties difficult. Biological silk production systems, which evolved independently in both silkworms and orb-weaving spiders, seem to converge, displaying several common spinning elements [6]. While, the spinning systems of both spiders and silkworms have common elements, the primary amino acid structures have specific differences in repeat motifs (Table 1) [7–9]. These differences in repeat motifs leads to a drastic difference in mechanical performance between spiders and silkworms. Importantly, silkworm silks are a single protein core (fibroin) coated with sericin; whereas, spider silks are a nanocomposite of two proteins. Nevertheless, the common thread between the spinning systems however have driven efforts to create an artificial material control system to produce silk-based, high-performance fibers.

**Table 1:** Spider and silkworm silk fiber structure repeat motifs.

Protein	Elastic β-Spiral GPGXX		Crystalline β-Sheet Ala-rich		3_10_-helix	Spacer	C-term	(GA)_n_GX	
	GPGGX	GPGQQ	(GA)_n_	A_n_	GGX	Unique	Unique	GAGAGS	GAGAGY
Spider Silk Dragline (MaSp1)			X	X	X		X		
Spider Silk Dragline (MaSp2)	X	X		X			X		
Silkworm Silk (Fibroin)						X		X	

Spider silk dragline proteins MaSp1 and MaSp2 repeat motifs giving structure of both mechanical strength and elasticity. Silkworm silks have simpler fiber structure with repeat motifs arranged to form large block chains interrupted by spacer sequences.

**Fig 1:**
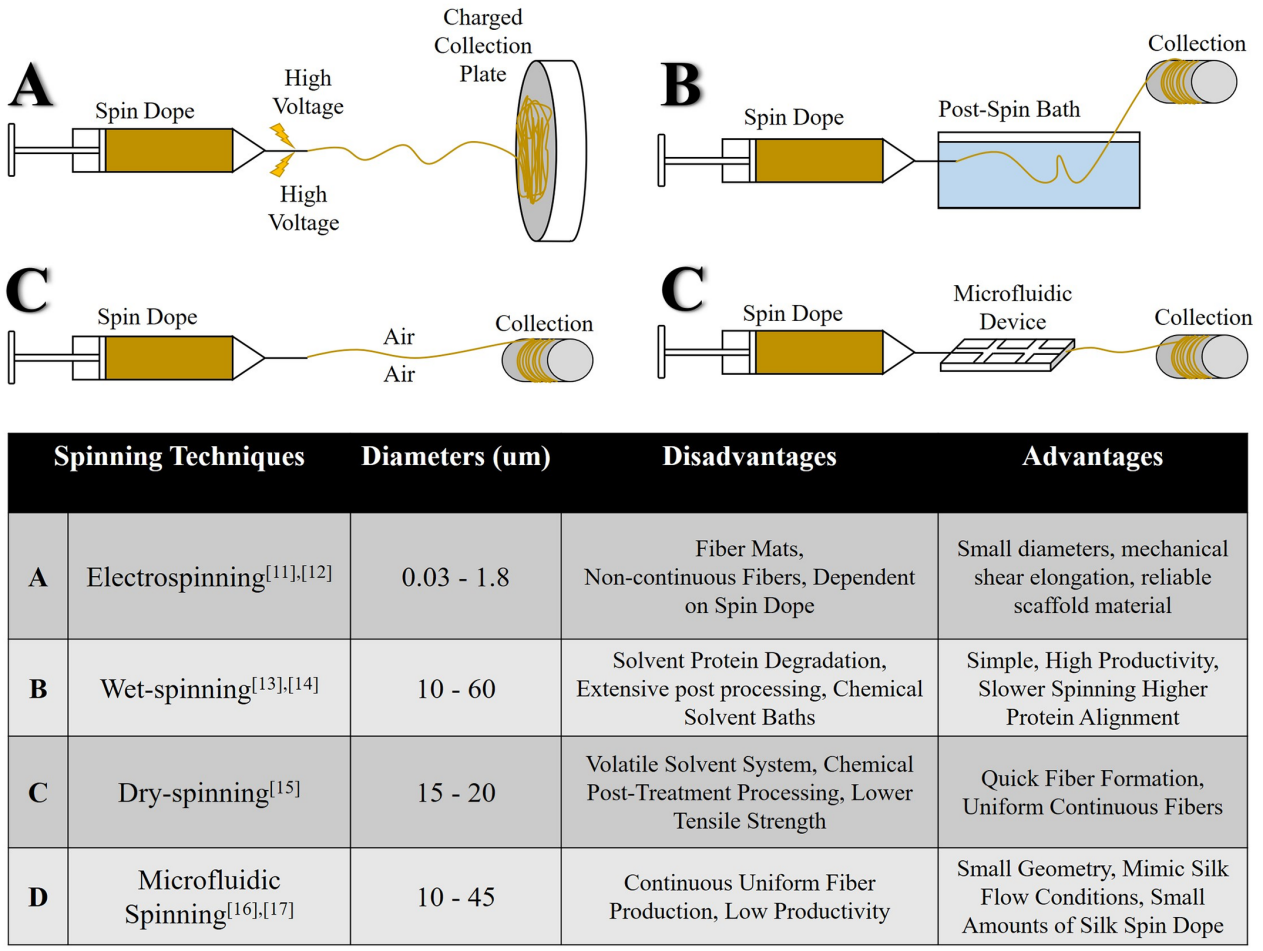
Multiple presented techniques to spin silk fibers synthetically. Popular silk spinning techniques including collected diameter value ranges with advantages and disadvantages of each technique.

Human manipulated fiber spinning systems should incorporate these common underlying principles, specifically (1) distinct zones of processing, (2) a combination of chemical and mechanical stimuli, and (3) an integrated process for spinning and drawing. The current simplified approach to silk spinning has led to the evolution of several different fiber spinning systems: electrospinning [10,11], wet-spinning [12,13], dry-spinning [14], and microfluidic spinning [15,16] (Fig 1). Although each of these systems have yielded fibers, none of these fibers exhibit the unique combination of strength and elasticity exhibited by natural silk fibers, potentially due to drastic differences in the process of fiber production (i.e., lack of chemical gradients and inconsistent mechanical shear). By neglecting shear flow and chemical gradients found in the natural spinning system, previously developed silk spinning systems have yielded inconsistent and inferior fibers.

**Fig 2:**
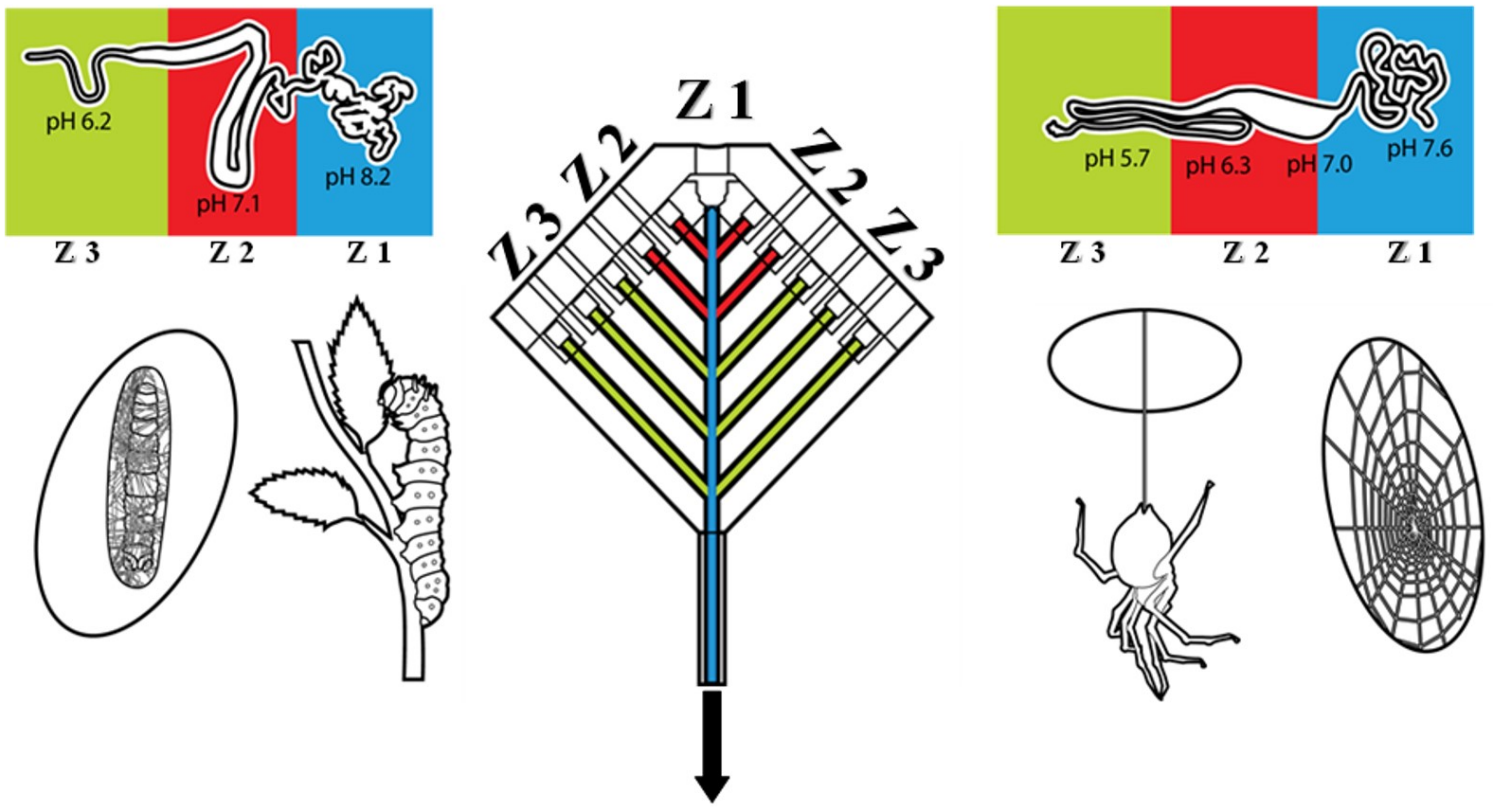
A biomimetic silk spinning system designed from *Nephila clavipes* and *Bombyx mori* silk glands. Schematic outlining the zones of fiber production in silkworms and spiders. Notice that the microfluidic system described in this manuscript mimics each zone. Zone 1 (**Z1**) mimics protein creation and beginning flow inlet to the system. Zone 2 (**Z2**) mimics the lumen of the gland through protein storage and beginning of biochemical stimulus. Zone 3 (**Z3**) mimics the duct region providing pH and ionic gradients as well as mechanical shear through hydrodynamic focusing.

Previous efforts to synthetically recapitulate the fiber’s mechanical properties used more simplistic processes, eliminating the complex interplay of chemistry, biology, and mechanical shear. To date, these artificial spinning systems cannot match the variable control of mechanical properties produced by spiders [17,18]. In both spiders and silkworms, silk fiber production can be divided into 3 zones: secretion (zone 1, **Z1**), storage (zone 2, **Z2**), and fiber production (zone 3, **Z3**) (Fig 2) [19]. In Z1, modified columnar epithelial cells lining the tail of the gland secrete proteins. These proteins are then translocated into Z2 for storage as a high viscosity protein solution [20]. Conversion of the solution to a solid fiber occurs primarily in Z3 by subjecting the protein solution to chemical stimuli (pH and ionic gradients) and mechanical shear flow, aligning amino acid elements to facilitate secondary and tertiary structures, thereby producing a physical fiber. To capture the natural formulation and controlled processing of the natural spinning system, it is necessary to emulate each element of the spinning process (Fig 2), including the controlled introduction of chemical stimuli (i.e., pH and ionic gradients) and integrated variable fluid flow for shear thinning. Both ionic and pH gradients will provide (1) electronegativity differentials along the spinning length to control protein alignment and (2) dehydration to facilitate hydrophilic/hydrophobic interactions and drive secondary structure formation. Although many synthetic fiber production systems strive to control these biochemical interactions, fluid flow within the system is often neglected.

Not only do current spinning systems disregard the three distinct zones but virtually all, except electrospinning, systems separate the spin and draw processes, requiring post-spin processing to improve mechanical properties and thin the fiber diameter [21–23]. Separating these two processes into spin, or coalescence of proteins, and draw, or shear thinning of the fiber, represents a fundamental departure from the natural spinning process resulting in inconsistent and mechanically inferior fibers. Furthermore, the extrusion, pushing action found in many artificial spinning systems subjects the protein solution to unnatural forces. To attain controlled diameters while combining the spin and draw process, fluid focusing or hydrodynamic focusing can be exploited. Hydrodynamic focusing uses fluid flow dynamics to narrow the central fluid stream within the microfluidic channel [24]. The use of fluid focusing provides the opportunity for controlled flow to be correlated with diameter [25]. Previous studies have coincided with the need for continued investigations of the dependence of the spinning process on fluidic flow spinning [26,27]. These studies investigate the strain dependence of the spinning process, which is a step towards the biomimetic processing. In these studies the use of a dual sheath fluidic flow focuses on a restricted geometry and prevents the ability to integrate chemical gradients. The current study presents (1) a preliminary correlation between silk spin dope fluid characteristics and the mechanical properties of the resulting fibers; (2) a microfluidic spinning system design that provides the ability to introduce a gradient of both chemical and mechanical stimuli [28,29]; and (3) integrated hydrodynamic focusing to eliminate the necessary post-spin processing found with other synthetic spinning systems. Ultimately, this combination of controlled fluid dynamics and mechanical shear allows predictable control of the output diameter of silk fibers.

## Materials and methods

### Solution preparation

Natural silks produced by the silkworm *Bombyx mori* supplied from Jiangsu Fu’an Cacoon and Silk Co. were degummed by boiling at 100°C in 0.05%wt Na_2_CO_3_ aqueous solution. Degummed silk was allowed to dry overnight. Dry degummed silk was directly dissolved as an 8% w/w concentration in a CaCl_2_ –formic acid solution for 3 hours at room temperature. This process was repeated to give 8, 10 and 12% w/w silk solutions. Alternatively, natural major ampullate silk produced by the spider *Nephila clavipes* (wild caught in Florida) was collected through forcibly silking in Brooks’ Lab at 20–30 rpm. The silk was dissolved in HFIP to produce an 8% w/v solution.

### Steady state rheology

Silk samples of concentration 8, 10 and 12%w/w were prepared for rheological analysis. Silk solutions were loaded into a TA ARG2 rheometer with a test plate size of 25mm. Each concentration was subjected to steady state shear at strain of 0.01%. The steady state shear continued to increase through a step range of 6.283–628.3 rad/s angular frequency.

### Fluid simulations

COMSOL Multiphysics simulation software was utilized to visualize and correlate fluid flow dynamics using custom microfluidic device channel dimensions generated using AutoCAD. Device geometry consisted of 5 channel inlets and 1 outlet channel. The length of main channel was 100 mm and the width ranged from 1 to 1.5 mm. Fluid shear changes were simulated using the COMSOL Laminar Flow Module by varying the fluidic rates and assessing stream line output. The main channel rate was held constant at 0.2ml/min while flow rates through the side channels were varied from 0.2 to 1.5 ml/min. The viscosity of solution in the side channels was that of isopropanol. The changes in fluid output dimension were measured at the last device intersection of **Z3** and diameter values were stored to correlate with corresponding fluid rates (Table 2). These reductions were correlated to physical diameters for predictability testing.

**Table 2:** Hydrodynamic focusing fluid simulations with initial and reduced fluid boundaries.

Fluid Simulations	Initial Fluid Boundary	Reduced Fluid Boundary
HF: 0.2 ml/min to 0.5 ml/min	1.5 mm	120 μm
HF: 0.2 ml/min to 1.0 ml/min	1.5 mm	74 μm
HF: 0.2 ml/min to 1.5 ml/min	1.5 mm	42 μm
HF: 0.2 ml/min to 5.0 ml/min	1.5 mm	4.4 μm

Fluid simulations investigating the change in fluid boundary in the microfluidic chip device caused by hydrodynamic focusing (HF).

### Hydrodynamic spinning

Silk solutions (i.e., 8, 10 and 12%w/w concentrations) used for rheometry were subjected to fluid flow within the custom microfluidic device. To control the flow rates through the side channels a 10-channel Hamilton syringe pump was used. The main channel was independently controlled with a single channel syringe pump. For each test condition, 1 ml of the silk spin dope was placed in a syringe and positioned in a single channel syringe pump and connected to the main channel of the microfluidic device. 1 ml of isopropanol was drawn in four separate syringes and placed in the 10-channel pump in parallel and connected to the side channels of the microfluidic device. Fluid flow rates were set based on data from the fluid flow simulations. Fiber diameters collected from each spinning test condition were measured under light microscopy on a Leica DMi8 microscope at 20x and 40x magnification and correlated to simulation data.

### Mechanical testing

Each 3cm length of fiber collected during spinning was secured to a specimen card for tensile testing using an Instron 5942 Micro-tensile testing apparatus on a 50-gram load cell. Each specimen was loaded at a rate of 2mm/min and tested until failure.

### Statistics

Fiber diameters are reported as an average (Excel 2016) of multiple independent replicates at each rate using hydrodynamic focusing (HF) and without hydrodynamic focusing (NHF). NHF0_2 spun at 0.2 ml/min (n = 3), NHF0_5 spun at 0.5 ml/min (n = 4), HF0_5 spun at 0.2 ml/min in the main channel Z1 and Z2 with 0.5 ml/min at Z3 (n = 9), HF1_0 spun at 0.2 ml/min in the main channel Z1 and Z2 with 1.0 ml/min at Z3 (n = 7), HF1_5 spun at 0.2 ml/min in the main channel Z1 and Z2 with 1.5 ml/min at Z3 (n = 4), HF5_0 spun at 0.2 ml/min in the main channel Z1 and Z2 with 5.0 ml/min at Z3 (n = 13). Mechanical testing of re-spun silk gathered from independent spinning at each rate NHF (n = 4), HF0_5 (n = 3), HF1_0 (n = 6), and HF1_5 (n = 3) are expressed as an average using a custom MATLAb code (v. R2016A) expressed with standard deviation of the mean of each individual sample size. Rheology of three different silk solutions (i.e., 8, 10 and 12%w/w concentrations) is expressed as an average (Excel 2016) (n = 3) for each solution.

## Results and discussion

Using a previously developed 3D printed, multichannel, microfluidic device (Fig 2), both chemical stimuli and gradient fluid flow can be integrated to directly mimic the natural gland system. Since silk protein alignment is naturally dependent on mechanical shear, laminar flow within Z2 and part of Z3 of the device maintains stable environmental conditions to resist premature fiber formation. Inhibition of premature fiber formation in the gland is thought to normally be a product of both laminar-like flow and the presence of the N- and C-terminal protein sequences that are often lacking in synthetic versions of the protein [30–32]. Thus, fiber formation as a product of shear thinning was exploited by creating mechanical shear conditions within the spinning system to control the fiber diameter.

**Fig 3:**
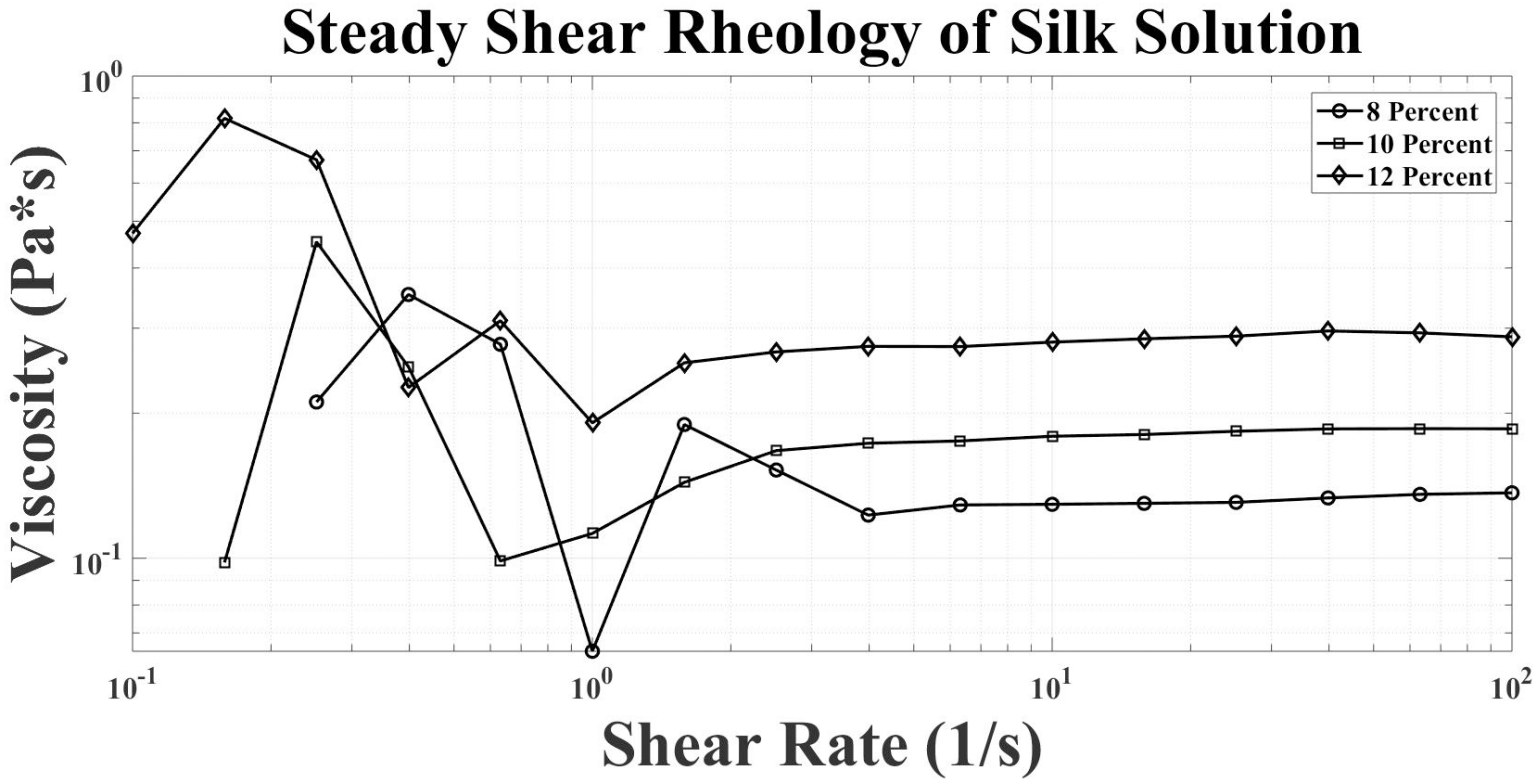
Characterized by rheology the silk system shows shear dependence during spinning for protein self-assembly. Rheological characteristics (steady shear) of three solutions with various silk protein concentrations are described. The viscosity trends follow a linear shear thinning decay transition and stabilization period showing silk proteins are shear dependent and react to increasing rate.

Repeatable fabrication of silk fibers relies on producing silk protein spin dopes with a protein concentration and viscosity necessary to facilitate protein coalescence, ultimately, driving the mechanical properties of the fiber. The natural concentration of silk proteins in the gland leads not only to a highly viscous solution but more importantly to a material that undergoes a phase transition under fluid shear rates. Dissolving natural silkworm silk in formic acid and calcium chloride at variable concentrations yielded viscoelastic spin dope solutions with characteristics similar to shear thinning polymers, i.e., lower viscosity with increased shear rate (Fig 3) [33–35].

This shear-thinning behavior, analogous to the natural spin dope, is thought to primarily be a function of the shear-dependent protein aggregation of the re-suspended silk protein used to make spin dopes. As shear increases, silk’s amino acid building blocks, which have been correlated to strength and elasticity, begin to align and secondary and tertiary structures are formed. The protein’s response to increased mechanical shear offers a plausible explanation for the dependence of fiber formation on shear rates. Although not contemplated in the context of mechanical shear, it is quite clear based on NMR [36,37] and X-ray diffraction studies that physical fiber formation is intimately tied to localized, motif-specific secondary structure, specifically, highly crystalline beta-sheet regions, which are stabilized by hydrogen bonding and hydrophobic/hydrophilic interactions [38]. Thus, from both rheological analysis and theoretical viscoelastic material behavior, it is clear that changing both the viscosity of the silk protein feedstock and the rate of shear flow will have a significant impact on fiber formation.

**Fig 4:**
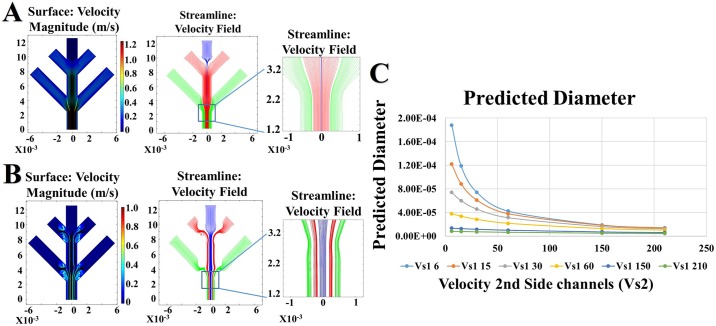
Fluid simulations provide predictable fiber diameters from hydrodynamic focusing shear rates. Fluid flow simulations that mirror the solution properties of the physical spin dopes were created. **A** Flow rates in the main center channel remained consistent while the side channels had increased fluid rates to simulate hydrodynamic focusing with axes representing geometry in micrometers (μm). **B** Flow rate introduced into the main center channel maintained consistent with rates of the side channels with axes representing geometry in micrometers (μm). **C** Predicted diameter outputs from hydrodynamic focusing fluidic rate simulations.

While rheological analysis provides insight into protein behavior under increasing fluidic shear within the artificial biomimetic device (Fig 2), in silico simulations in which fluid flow rates can be altered and Z1 fluid boundary conditions (i.e., contact between the solution and the plastic resin wall of the channel) restricted, instigating increased fluid shear, provides a valuable tool to predict fiber formation. Fluid simulations were designed to understand the impact of altering fluid viscosity and flow rates on fiber diameter (Fig 4). Using the viscosities obtained from rheometry and from the literature, repeated in silico trials to alter the rate of fluid flow were able to establish a mathematical relationship between the rate of fluid flow and the diameter of the artificial re-spun fiber (Fig 4, Eq (1)).

$$D=0.0001e^{-0.014(\frac{d*\tau }{\mu })}$$ (1)

The relationship of fiber diameters (**D**) collected from both simulations and benchtop hydrodynamic focused spinning show an exponential decrease corresponding with the width of the microfluidic channel **d**, fluid shear stress τ and solution viscosity μ. Importantly, while native *Nephila clavipes* fibers have an average diameter of 3–7 μm, previous studies have established that high strength fibers have smaller diameters and more elastic fibers having larger diameters [39–41]. Thus, synthetic production of high-performance fibers with tailored mechanical properties requires the ability to predictably and consistently modify the fiber diameter. Fluid flow simulations, which established this relationship, were subsequently replicated on the bench and used to spin physical fibers in an analogous process.

Attaining consistent, small diameter fibers based strictly on the physical dimensions of the system is challenging at best and often insurmountable. Thus, previous efforts to create smaller diameter fibers have been focused on the need to 1) limit the boundary conditions of the spinning environment and 2) alter the post spin draw ratio. Post spin processing, in addition to introducing inconsistency, has yet to yield a fiber that can rival the mechanical strength of its natural counterpart.

**Fig 5:**
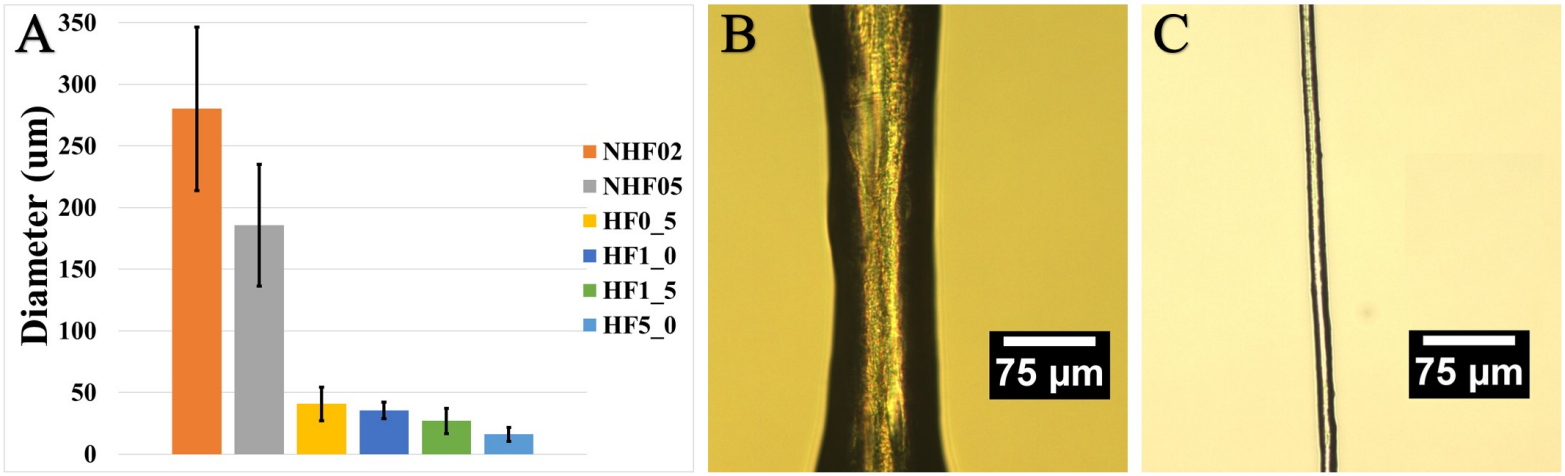
Increasing fluid shear through biomimetic spinning system by hydrodynamic focusing can reduce fiber diameter. The relation and depiction of spin rate to fiber diameter output **A**. Fiber produced using no hydrodynamic focusing **B** corresponding to NHF0_2. Fiber spun using hydrodynamic focusing **C** corresponding to HF5_0.

To investigate the impact of smaller boundary conditions due to fluid focusing within the microfluidic-spinning device and isolate the impact of spinning on the mechanical properties of the fiber, both silkworm silk and spider silk were dissolved and re-spun. Using a ratio of fluid flow rates and solution viscosity, the diameter of the center channel fluid (i.e. silk) could be narrowed or focused to yield small diameter fibers (Fig 5). Spinning with a variable fluid rate provides shear gradients that restrict the boundary condition of the silk spin dope, effectively narrowing the spin dope fluid stream. This focusing leads to a fluidic draw that occurs naturally as the spin dope flows through the intersection of the side channels that output a lower viscosity fluid (i.e. isopropanol) at a higher rate. Traditionally, in synthetic silk spinning systems, post spin draw solvent baths that facilitate dehydration are used to promote secondary structure as the fibers are being spun (i.e., methanol, isopropanol (IPA)) [42]. Nevertheless, it is possible that the use of IPA compromises the mechanical properties of the fiber due to rapid dehydration. PEG infused IPA may facilitate a better protein alignment and may be tried in the future. Localized shear stress acting on the spin dope at the channel junction facilitates protein alignment and likely promotes beta-sheet alignment and fiber formation. Using flow to physically move or draw the fiber through the device, subjects the fiber to more natural mechanical forces, leading to more uniform, consistent fibers, a distinct advantage of the current system.

By altering the fluid flow rate of the outer side channels in Z3 to 0.5 ml/min (HF0_5), 1.0 ml/min (HF1_0), 1.5 ml/min (HF1_5), and 5.0 ml/min (HF5_0)), while holding the main channel Z1 at constant rate (0.2 ml/min), fibers with diameters within 15% of the natural fiber diameter were spun using hydrodynamic focusing (HF). As predicted by fluid flow simulations, the smallest diameters attained ranged between 5–7 μm; however, this pushed the limits of the syringe pump. Diameters could reliably be obtained between 10–12 μm at HF5_0. Conversely, in the absence of hydrodynamic focusing (NHF) (i.e., all fluid flow rates being the same), fiber diameters were more than 20x larger, averaging 275–280 μm.

**Fig 6:**
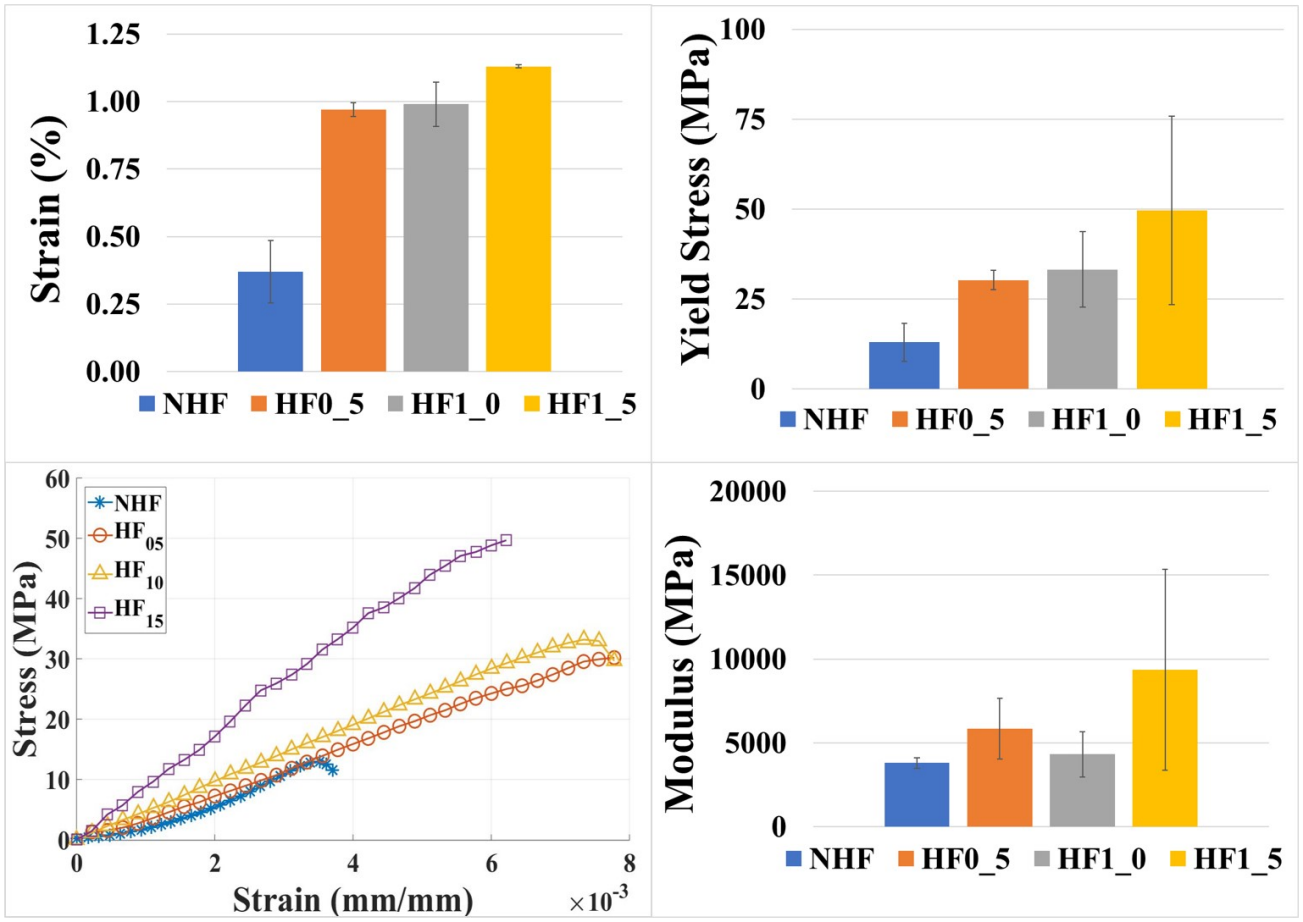
Hydrodynamic focusing rates affect fiber diameter allowing for control of mechanical performance. Mechanical testing revealed a gradual increase in tensile strength as higher focusing rates were set in the system. Both yield stress and breaking strain were gradually improved through the controlled decrease in fiber diameter. Young’s modulus on the other hand had shown to stay steadily similar between each test. Modulus is dependent on the molecular structure and characteristics of the material.

Tensile testing of the re-spun fibers with no hydrodynamic focusing and with hydrodynamic focusing displayed increasing mechanical strength with decreasing diameter and increasing fluid shear (Fig 6, Table 3). The resulting change in yield stress and breaking strain correspond to the dependency of fiber geometry. Interestingly, young’s modulus remains relatively consistent showing little to no change due to the change in fiber diameter [43]. This is attributed to the consistency of material characteristics and spinning of the silk fibers. Additionally, the modulus seems to be governed by the molecular structure of the fiber and is relatively independent on the characteristics of the material [43]. Nevertheless, despite the controlled fluidic shear spinning, the resulting fibers are still mechanically inferior to the natural silk fibers, suggesting the needed integration of chemical gradients in the spinning system [20,44–47].

**Table 3:** Comparison of mechanical performance of synthetically spun silk fibers using hydrodynamic focusing.

Fiber Type	Yield Stress (MPa)	Breaking Strain (%)	Modulus (GPa)
NHF	13.0 ± 5.35	0.4 ± 0.12	3.8 ± 0.32
HF0_5	30.2 ± 2.70	1.0 ± 0.03	5.8 ± 1.80
HF1_0	33.2 ± 10.48	1.0 ± 0.08	4.3 ± 1.35
HF1_5	49.7 ± 26.15	1.1 ± 0.01	9.4 ± 6.00
Natural Silkworm	600	18	17
Natural Spider silk (Dragline)	1500	27	10

Yield stress and breaking strain shows an increasing trend as fibers are spun using higher rates of hydrodynamic focusing resulting in smaller diameters. Still the hydrodynamic fibers are inferior to natural silk fibers due to the lack of chemical gradient spinning.

## Conclusion

Despite decreased diameters, the strength and elasticity of the re-spun fibers are still no match for their natural counterpart. However, this study demonstrates the ability of hydrodynamic focusing in the absence of gravimetric pull to narrow the diameter of the fiber in a predictable way and increase the mechanical performance. Additionally, this study reveals more precisely the relationship between fluid viscosity and shear rate leading to the derivation of a predictive equation for the diameter. Spinning at specific rates yields fibers with diameters that matched the in-silico predictions, leading to a complete spinning system that can provide fibers to rival the diameters of the natural silk produced by both silkworms and spiders. Although closely approximating the diameter of the natural fiber did not fully recapitulate its mechanics, future efforts to integrate chemical stimuli (e.g., pH and ionic gradients) are predicted to increase protein alignment and improve the fiber’s mechanical performance.

Nevertheless, the importance of producing fibers with a predictable diameter via hydrodynamic focusing cannot be understated. By focusing the fluid stream within the device, the concept of hydrodynamic focusing can constrict the boundary conditions of silk spin dope fluid flow and yield fibers with smaller diameters. Future efforts to integrate elements of pH and ionic gradients in unison with fiber focusing to control the assembly of beta-sheet and helical amorphous regions are expected to further improve the mechanical performance of synthetic fibers.
